# A novel integrative risk index of papillary thyroid cancer progression combining genomic alterations and clinical factors

**DOI:** 10.18632/oncotarget.15128

**Published:** 2017-02-06

**Authors:** Qing Cheng, Xuechan Li, Chaitanya R Acharya, Terry Hyslop, Julie Ann Sosa

**Affiliations:** ^1^ Department of Surgery, Duke University Medical Center, Durham, NC 27710 USA; ^2^ Department of Biostatistics and Bioinformatics, Duke University Medical Center, Durham, NC 27710 USA; ^3^ Department of Medicine, Duke University Medical Center, Durham, NC 27710 USA; ^4^ Duke Cancer Institute, Duke University Medical Center, Durham, NC 27710 USA

**Keywords:** papillary thyroid cancer, disease progression, genomic alterations, clinical and pathologic factors, risk index model

## Abstract

Although the majority of papillary thyroid cancer (PTC) is indolent, a subset of PTC behaves aggressively despite the best available treatment. A major clinical challenge is to reliably distinguish early on between those patients who need aggressive treatment from those who do not. Using a large cohort of PTC samples obtained from The Cancer Genome Atlas (TCGA), we analyzed the association between disease progression and multiple forms of genomic data, such as transcriptome, somatic mutations, and somatic copy number alterations, and found that genes related to FOXM1 signaling pathway were significantly associated with PTC progression. Integrative genomic modeling was performed, controlling for demographic and clinical characteristics, which included patient age, gender, TNM stages, histological subtypes, and history of other malignancy, using a leave-one-out elastic net model and 10-fold cross validation. For each subject, the model from the remaining subjects was used to determine the risk index, defined as a linear combination of the clinical and genomic variables from the elastic net model, and the stability of the risk index distribution was assessed through 2,000 bootstrap resampling. We developed a novel approach to combine genomic alterations and patient-related clinical factors that delineates the subset of patients who have more aggressive disease from those whose tumors are indolent and likely will require less aggressive treatment and surveillance (*p* = 4.62 × 10^–10^, log-rank test). Our results suggest that risk index modeling that combines genomic alterations with current staging systems provides an opportunity for more effective anticipation of disease prognosis and therefore enhanced precision management of PTC.

## INTRODUCTION

It is anticipated that more than 64,300 Americans will be diagnosed with thyroid cancer in 2016 [1]; nearly 90% will have papillary thyroid cancer (PTC). PTC is the fastest increasing cancer in men and women in the U.S.; it is already the most common cancer among women ≤ 35 years, and the incidence and aggressiveness of this malignancy increase with patient age. With the aging of the U.S. population, it is anticipated that the observed increase in incidence of nearly 3-fold over the past 30 years will only continue [2]; in part, this is due to increased use of diagnostic imaging modalities, such as CT, PET, MRI, and ultrasound [3]. Almost the entire change has been attributed to an increase in the incidence specifically of PTC that has been observed across all age groups [4], with an increase of more than 150% over the last decade alone.

Fortunately, PTC is usually indolent and can be effectively treated with surgical resection, often combined with radioiodine therapy. Although treatment is usually curative, with an overall 5-year survival rate of about 95%, survival drops precipitously in the setting of locally advanced, metastatic, and/or radioiodine-refractory disease; indeed, the five-year survival rate for patients with Stage IV disease is just 50%, and thyroid cancer-related mortality among men has increased at a higher rate in the U.S. than for any other cancer. Recurrence rates have been reported to be as high as 20–30%, and more than 10% of patients die as a result of disease progression [5, 6]. Some patient-related factors such as advanced age at diagnosis, larger tumor size, cervical lymph node metastases, extrathyroidal extension, and lymphovascular invasion have been associated with an increased risk of disease progression at a population level, but they lack the precision to perfectly tailor surveillance and treatment strategies and anticipate prognosis [7].

In thyroid cancer, as in other malignancies, molecular markers have emerged as an adjunct that can be used to potentially guide precision medicine. Thyrotropin (TSH) is the major growth factor and regulator of the thyroid, and serum TSH concentration is associated with the risk of cancer in a thyroid nodule [8, 9]. Serum thyroglobulin (Tg) level doubling time is used to anticipate the rate of progression (i.e. the shorter the doubling time, the faster the rate of progression) [10]. However, serum Tg has limited sensitivity for the detection of micro-metastases, and it is not found to be elevated in a substantial minority of patients with advanced disease and in those patients who have anti-thyroglobulin antibodies [11].

Identification of somatic mutations has been widely investigated as an adjunct that may help resolve the diagnostic uncertainty. Testing of BRAF V600E has been extensively studied in relation to predicting risk of recurrence of PTC and mortality [12]; results to date have been inconsistent [13]. The association between BRAF V600E and negative prognostic clinicopathologic features alone is unlikely to alter postoperative treatment decisions when histologic tumor variables also are available. Commercially available genomic tests such as the Veracyte Afirma^®^ gene expression classifier, Asuragen miRInform™ thyroid panel, and ThyroSeq offer claims of improved diagnostic sensitivity and specificity of fine needle aspiration (FNA) for evaluation of thyroid nodules and the risk of thyroid cancer in the setting of indeterminate cytology. However, none of these panels provide the opportunity to predict or monitor the progression of PTC. To this end, novel baseline prognostic factors that could be added to risk stratification algorithms are required.

The Cancer Genome Atlas (TCGA) data provide a unique opportunity to enable different and potentially complementary forms of analysis of cancer phenotypes given the comprehensive nature of the datasets generated in this effort. In this study, using multiple forms of genomic measurement of the same set of PTC samples obtained from TCGA, a subset of genes was identified for which expression, mutations or copy number alterations in primary PTC samples were associated with PTC progression. We demonstrated a risk index model strategy which combines genomic alterations with current staging systems, thereby providing an opportunity to delineate the subset of patients who have more aggressive disease from those whose tumors are indolent and likely will require less aggressive treatment and surveillance.

## RESULTS

To characterize the features of primary PTC that were associated with disease progression, we down-loaded genomic data for 505 primary thyroid tumor samples from TCGA. The clinical and pathologic characteristics of these samples are summarized in Supplementary Table 1. The median follow-up of the cohort was 30.6 months (range, 1–169.4 months). Among these samples, 60 (8.4%) had disease progression by the time of last follow-up, and the median duration between diagnosis and the first progression event was 17.4 months (Supplementary Figure 1).

### PTC progression-associated oncogenic signaling

**Figure 1:**
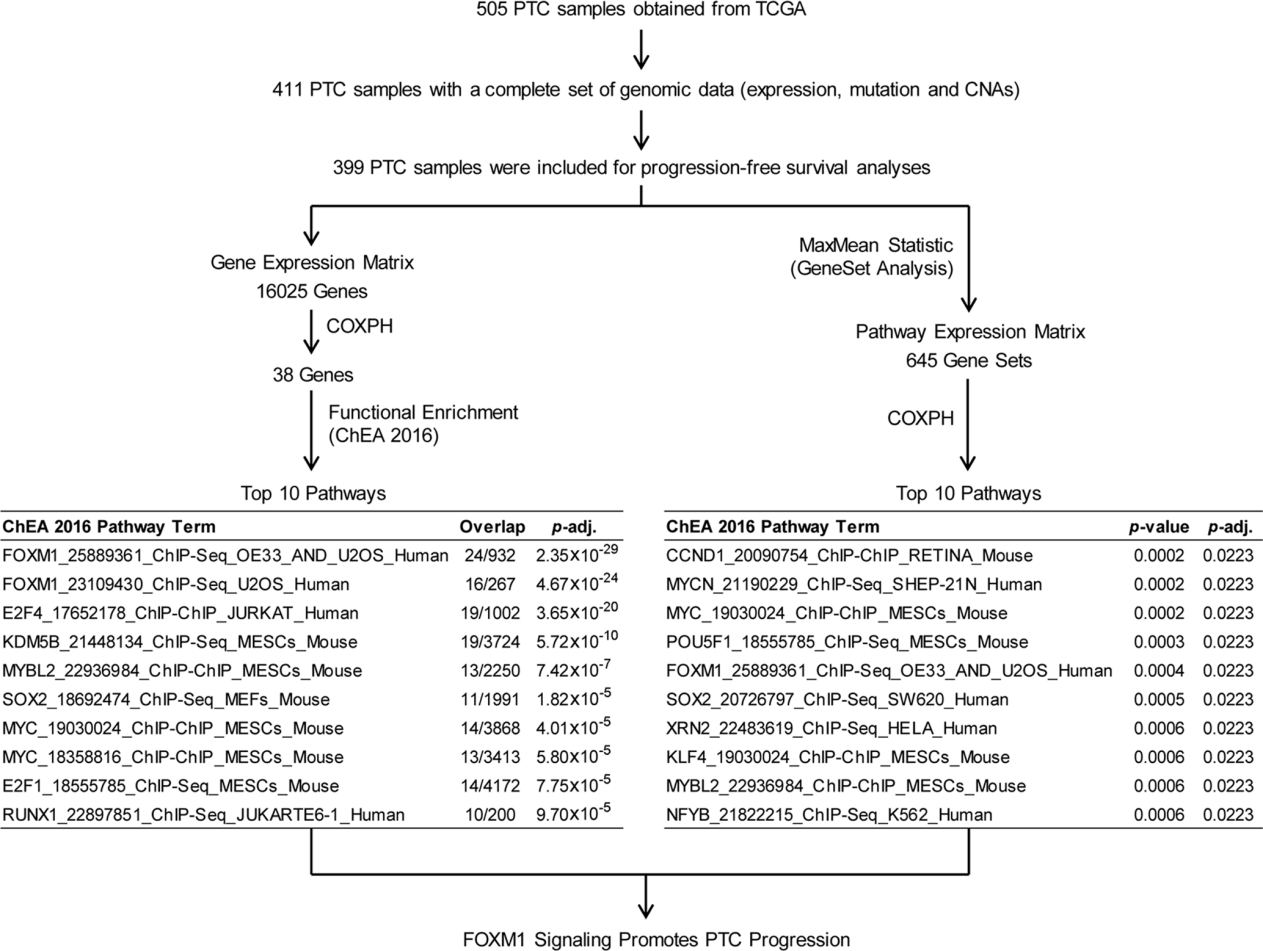
Pathway analyses of PTC progression associated signaling Both Functional enrichment and GeneSet analyses were conducted to identify signaling pathways that were associated with PTC progression.

**Figure 2:**
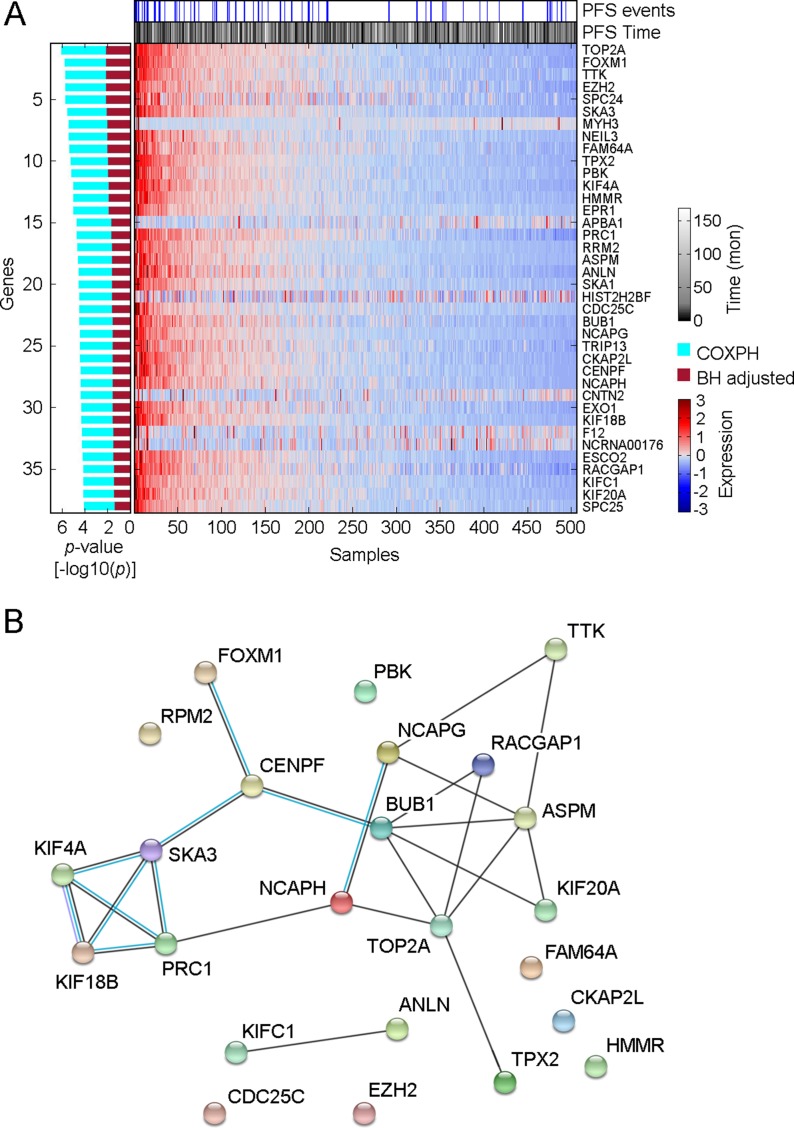
Distribution of genes whose expression was significantly associated with PTC progression (**A**) Heatmap shows the expression distribution of 38 PTC progression-associated genes among 505 THCA samples. The association between gene expression and PTC progression was assessed by COXPH survival analysis using 399 PTC samples for which a complete set of clinical data was available. The *p-value* of COXPH survival analysis was further adjusted by the Benjamini-Hochberg (B–H) procedure. (**B**) Protein-protein interaction plot obtained using STRING (version 10.0) for the speci_ed proteins in FOXM1 pathway. Black lines connecting the nodes indicate predicted interactions based on observed patterns of simultaneous expression of genes while the blue lines are known interactions from protein databases.

To assess oncogenic signaling that was associated with PTC progression, we performed both GeneSet analysis and Functional Enrichment analysis using PTC transcriptomes measured by RNAseq (Figure 1). Because preoperative medical therapy (e.g. radioactive iodine or small molecule therapy) could affect the gene expression profile of the primary tumor, we focused on those PTC samples that did not receive neoadjuvant treatment. Since PTC is usually treated initially with surgery, only 4 samples were lost due to this exclusion criterion. To identify PTC progression-associated gene expressions and pathway signaling that were independent of clinical confounding factors, we conducted a series of Cox Proportional-Hazards Regression (COXPH) survival analyses. First, we generated a gene expression matrix of 16205 genes. After further adjusting *p*-values from the COXPH by the Benjamini-Hochberg Procedure, we identified 38 genes whose expression was significantly associated with PTC progression (BH Adjusted *p-value* < 0.05, Figure 2A, Supplementary Table 2). Functional enrichment analysis revealed 5 signaling pathways that were highly enriched in high risk PTC (Adjusted *p-value* < 1 × 10*^–6^*, Figure 1). Particularly, 24 of these 38 genes were related to the FOXM1 signaling pathway (Figure 2B, Supplementary Table 3).

Second, sample-level enrichment scores for a total of 645 gene-sets/pathways were generated from gene expression data using gene set analysis (GSA) software. Statistically significant pathways were identified from a COXPH model that accounted for several clinical variables as described in the methods section. Our method identified 5 pathways (*p-value* < 0.0005, COXPH, Figure 1) that were significantly associated with PTC progression, a list that includes FOXM1 signaling pathway. In thyroid tumors, it has been reported that FOXM1 promotes the pathogenesis of PTC [14] and the invasive phenotype of anaplastic thyroid carcinoma [15]. In this study, we found that FOXM1 signaling was significantly associated with PTC progression, regardless of tumor stage and histological subtypes, indicating that the expression of genes related to FOXM1 signaling in the primary tumor might confer the potential for PTC progression.

### Recurrently altered genes and their association with PTC progression

**Figure 3:**
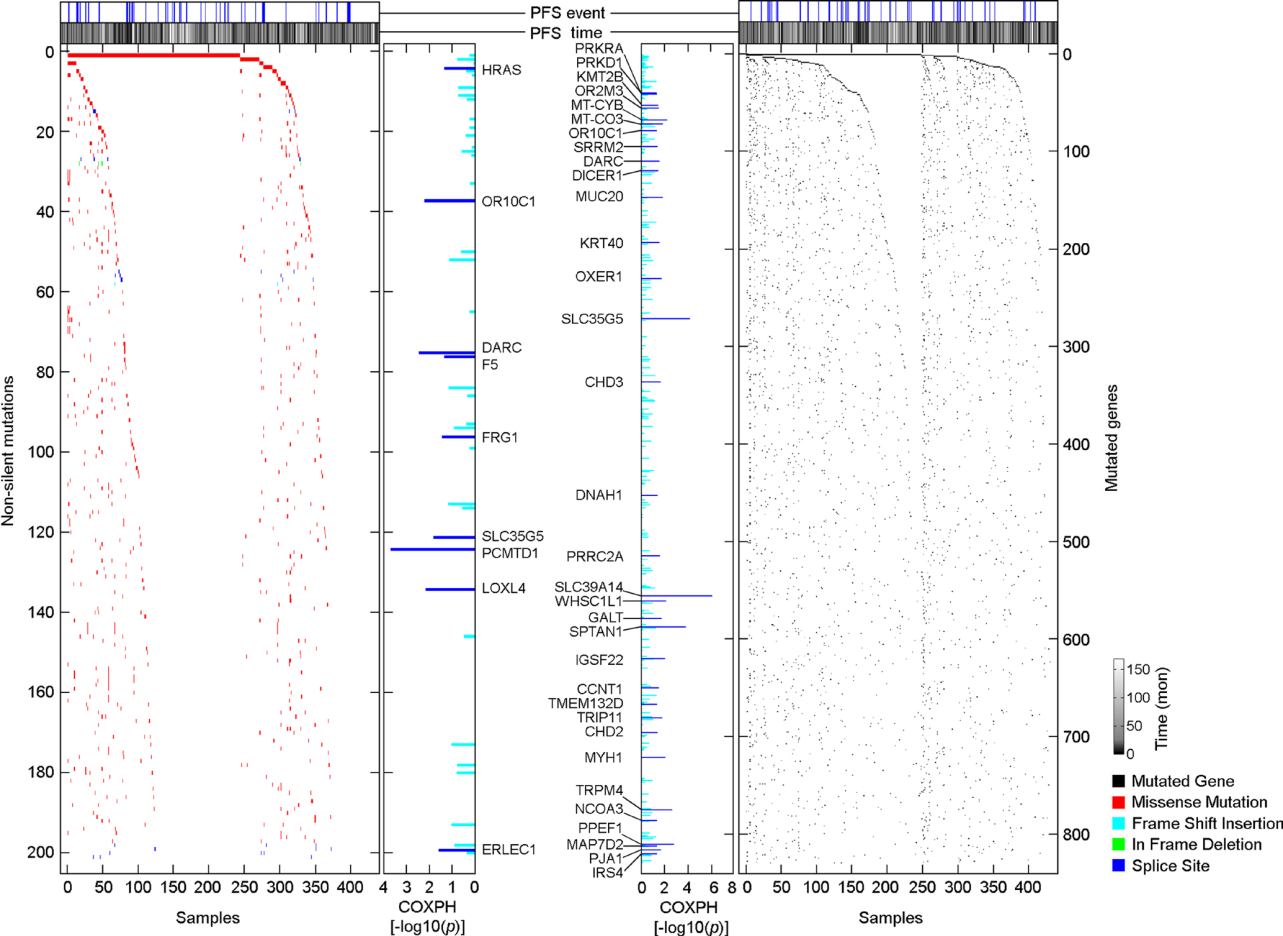
Distributions of PTC progression-associated, non-silent somatic mutations and mutated genes Scatterplots show the distribution of NSSM and mutated genes among 430 primary PTC samples. The association of non-silent mutations or mutated genes and PTC progression was assessed by COXPH survival analysis using 399 PTC samples for which a complete set of clinical data was available.

Somatic mutations in 424 PTC samples were measured by whole exome DNA sequencing. Among 9996 non-silent somatic mutations [NSSM, including non-silent Somatic single nucleotide variants and InDel (insertions or deletions of a few basepairs)], we determined that 201 (2%) NSSM were recurrently detected in 3 or more samples (Supplementary Figure 2). Because individual genes can be affected by multiple NSSM, we further measured the distribution of mutated genes that were affected by NSSM. Among 6437 mutated genes, we found that 829 (12.9%) mutated genes were recurrently detected in 3 or more samples (Supplementary Figure 2). We performed a series of COXPH progression-free survival analyses, and identified 9 recurrently detected NSSM and 33 recurrently detected mutated genes that were significantly associated with PTC progression, and the associations were independent of clinical confounding factors (*p <* 0.05, COXPH; Figure 3, Supplementary Tables 4 and 5). Oncologic pathway analyses revealed that PTC progression-associated mutated genes affect multiple molecular functions, biological processes, and cellular components of PTC, such as protein binding, cellular response to stimuli, and cell-cell junctions, by which they might be involved in PTC progression (Supplementary Table 6).

Consistent with a previous study published by TCGA research network [16], the most commonly mutated gene that was affected by NSSM in PTC was BRAF (57.7%), followed by FLG (9.3%), NRAS (7.9%), and TTN (7.2%). However, none of these commonly mutated genes were significantly associated with PTC progression (Figure 3), as a large proportion of patients with these commonly detected mutated genes did not experience disease progression. On the other hand, rare mutated genes were highly enriched in the primary tumors obtained from patients who experienced disease progression after initial treatment (Figure 3, Supplementary Tables 4 and 5). Our finding that only a subset of patients with BRAF mutations are at high risk of disease progression suggests that the role of mutated BRAF in promoting PTC progression may require secondary genomic alteration to modify the process. Although these rare NSSMs or InDels only affect a small fraction of the overall patient population, the collection of these rare mutations could be clinically significant in predicting disease outcome.

**Figure 4:**
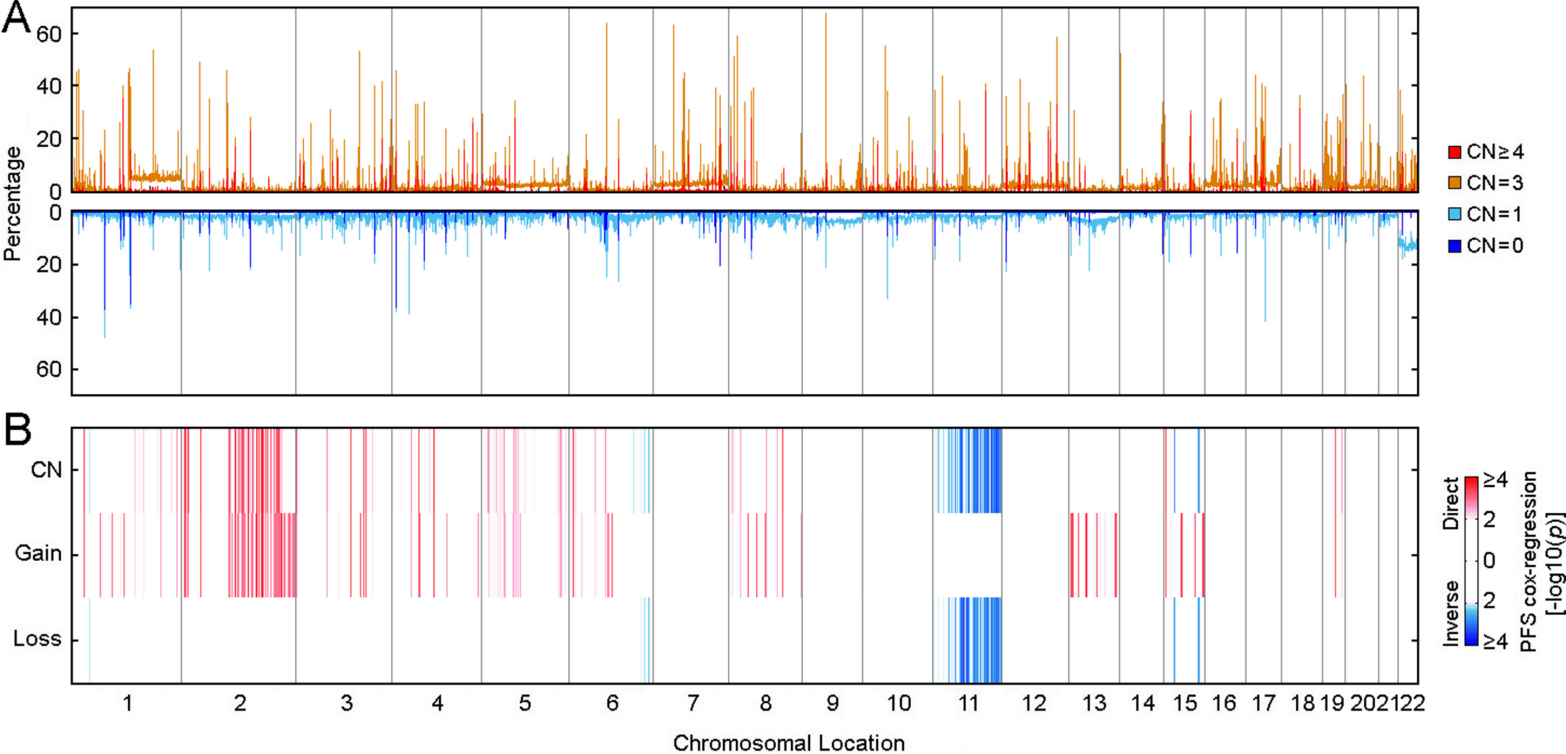
Distribution of somatic copy number alterations and their association with PTC progression (**A**) Genome scans for somatic CNAs distribution among 489 THCA samples. (**B**) Genome scans for PTC progression-associated somatic CNAs. Association between somatic CNAs and PTC progression was assessed by Cox-regression survival analyses, and the Heatmap shows the distribution of *p*-values. “CN” represents the Cox-regression analysis performed using copy numbers (0, homozygous deletion; 1, hemizygous deletion; 2, normal copy number; 3, low level amplification; 4, high level amplification). “Gain” represents the Cox-regression analysis focused on copy number gain (2, High level amplification; 1, Low level amplification; 0, all the others). “Loss” represents the Cox-regression analysis focused on copy number loss (-2, Both alleles deletion; 1, LOH; 0, all the others). Coefficients were examined to determine if copy number per se was a direct (higher copy number was associated with compromised outcome) or inverse (higher copy number was associated with better outcome) association.

Another form of genomic alteration is somatic copy number alterations (CNAs) that can quantitatively affect clinical phenotype and disease outcome [17, 18]. Cox-regression progression-free survival analysis revealed 95 copy number regions that were significantly associated with PTC progression (*p <* 0.0001, Figure 4), and 44 of these regions were independent of clinical confounding factors (*p <* 0.05, COXPH; Supplementary Table 7). Among these 44 identified copy number regions, 35 of 38 copy number gain regions that were associated with PTC progression resided within chromosome 2q, and all PTC progression-associated LOH or deletion regions were located on chromosome 11q (Supplementary Table 7). It has been reported that gain in 2q and loss in 11q is involved in the progression from benign follicular adenomas to follicular carcinomas [19, 20], while gain in chromosome 2q also is linked to tumorigenesis in non-medullary thyroid cancer [21]. Oncologic pathway analyses revealed that genes within those PTC progression-associated SCNA regions were related to protein or DNA binding, catalytic activity, metabolic process and response to stimulus, as well as intracellular or extracellular region parts (Supplementary Table 8).

### An integrative risk index of PTC progression

**Figure 5:**
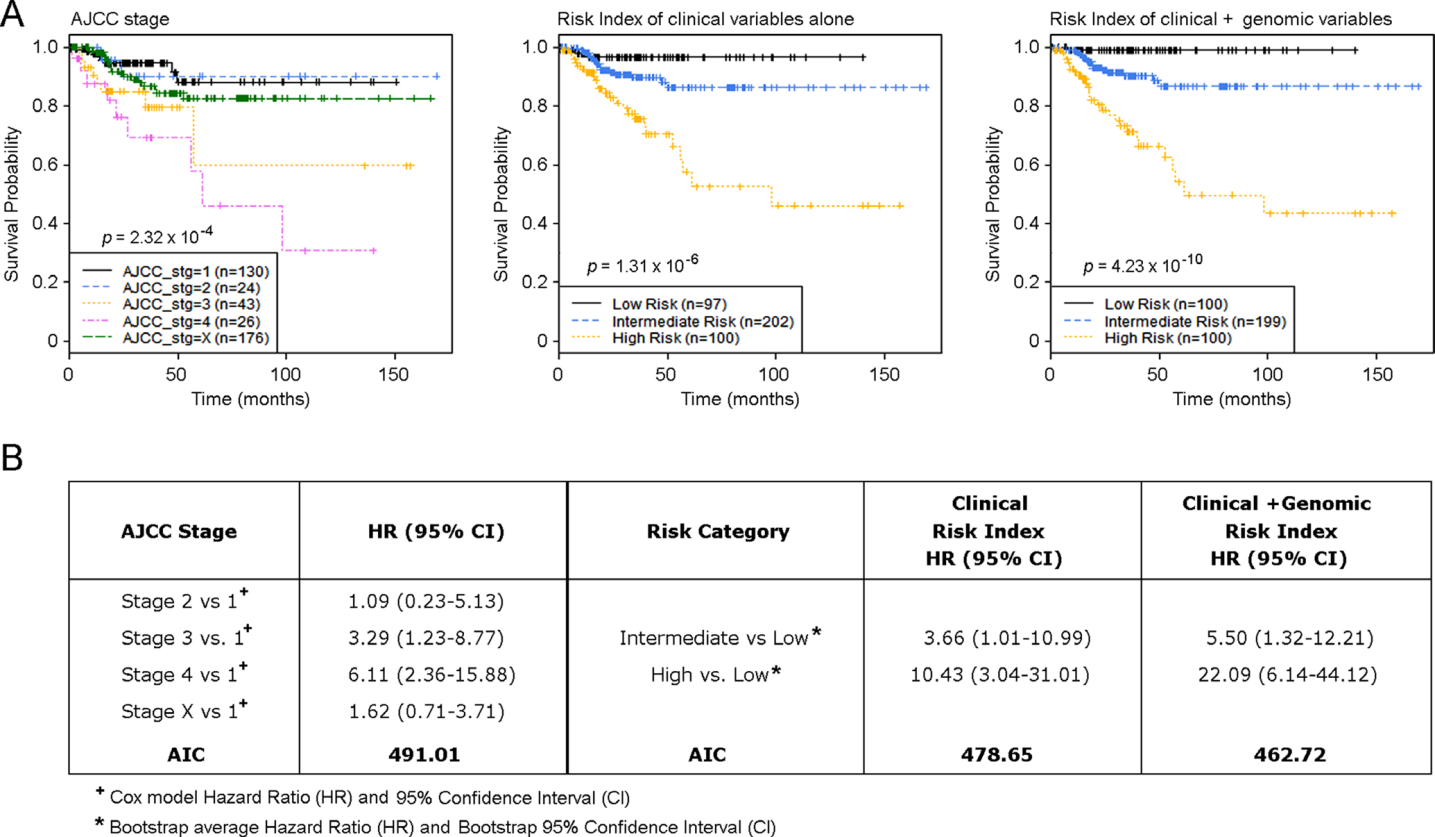
Risk index model defines primary PTC samples with a distinct risk of disease progression (**A**) Kaplan-Meier estimates of PTC progression-free survival of AJCC stages, risk model of clinical factors alone, and risk index model using combined clinical and genomic factors. (**B**) Hazard Ratio (HR) and AIC summary for groupings based on AJCC and Risk Index models.

As described in the Methods section, we developed a CRI (Clinical Risk Index) model and the C+GRI (Clinical plus Genomic Risk Index) model of PTC progression using elastic net regression and a leave-one-out cross validation procedure, and carried out internal validation of the model using 2,000 bootstrap resampling (Supplementary Tables 9 and 10). Risk index modeling that combines genomic alterations with clinical and pathological factors provides more effective prediction of PTC disease progression compared to AJCC staging and the predictive model using clinical factors alone (Figure 5), based on a comparison of AIC (Akaike Information Criterion). The AIC for the AJCC stage model is 491.01, for the CRI model is 478.65, and for the C+GRI model is 462.72. The lowest AIC for the C+GRI model indicates superiority of this model over the comparator models. The bootstrap average AUC for the CRI model ranged from 0.67 to 0.68 and for the C+GRI model from 0.72 to 0.74. The average bootstrap AUC difference, (d = AUC_C+GRI_–AUC_CRI_) for 1, 3, and 5 years, with 95% bootstrap confidence intervals, are 0.054 (0.009–0.107), 0.057 (0.008, 0.111), and 0.057 (0.009, 0.111), demonstrating that the C+GRI model provides a significant improvement in predicting risk over the proposed CRI model (Supplementary Table 11).

**Figure 6:**
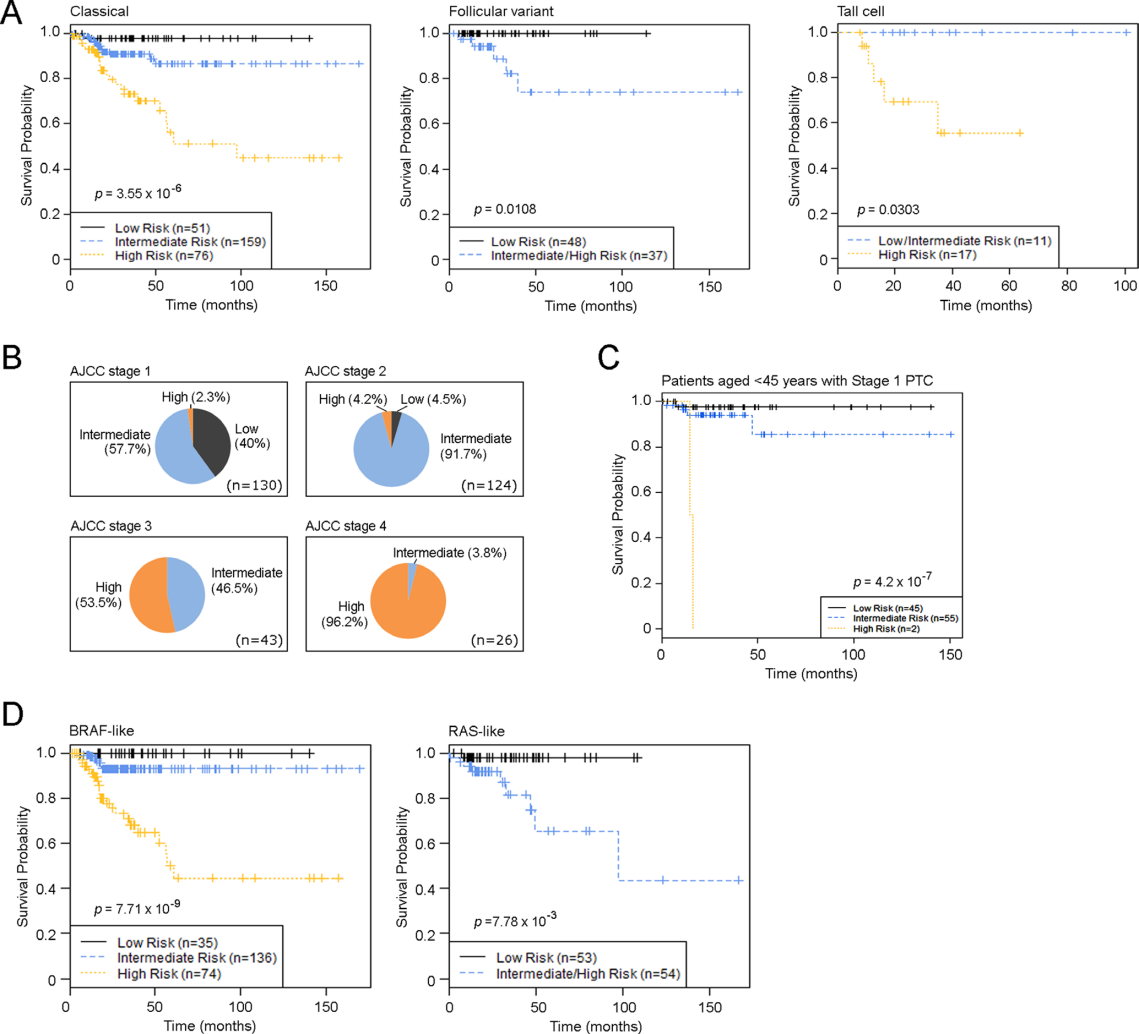
PTC Risk Index classification in PTC subtypes and in different disease stages (**A**) Kaplan-Meier estimates of PTC progression-free survival of the novel risk index model using combined clinical and genomic factors in different PTC histological subtypes. (**B**) Distribution of risk groups in different AJCC stages. (**C**) Kaplan-Meier estimates of PTC progression-free survival of the novel risk index model among patients aged < 45 years with M0 PTC. (**D**) Kaplan-Meier estimates of PTC progression-free survival of the novel risk index model in TCGA-defined PTC genomic subtypes. *p*-values were calculated using the log-rank test. Tick marks indicate patients whose data were censored by the time of last follow-up.

The C+GRI model estimated progression-free survival for patients in the Low risk group to be 97.8–100% for the entire dataset, as well as within each individual PTC histologic subtype, such as the Classical, Follicular, and Tall cell variants (Figure 6A). We found that over 90% of AJCC Stage 2 PTC patients had an intermediate risk of progression, while 46.5% of AJCC Stage 3 patients had an intermediate risk of PTC progression, indicating that a large proportion of patients with PTC in AJCC Stage 3 had a similar disease outcome to those with AJCC Stage 2 status. This raises the question of whether nearly half of Stage 3 PTC patients indeed need aggressive and potentially morbid treatment if their outcome is likely to be similar to that of patients with Stage 2 disease (Figure 6B). While the majority of patients with AJCC Stage 1 PTC experienced a low risk of disease progression, a small fraction carried a genomic phenotype similar to that of patients with later stage tumors (Figure 6B). For example, among patients who were aged < 45 years with M0 (AJCC Stage 1) disease, our risk index model using genomic alterations combined with clinical and pathological factors identified a small fraction of patients in a High risk group who had disease progression within 2 years of their diagnosis (Figure 5).

A recent study from TCGA research network demonstrated two main drivers of PTCs BRAF (primarily V600E) and RAS mutations - and defined 97% of PTC into two major subtypes, BRAF-like and RAS-like tumors [16]. We applied our C+GRI model to a TCGA defined BRAF-like and RAS-like PTC classification system, and defined high-risk samples as BRAF-like or RAS-like (Figure 6). The results in Figure 5 demonstrate that the C+GRI model provides new information outside existing known prognostic factors (eg stage), and also defines high risk groups that may be amenable to therapeutic options targeting these mutation subtypes.

## DISCUSSION

In this study, we demonstrate a novel risk index modeling approach that utilizes clinical and pathologic factors as well as genomic alterations in an effort to augment traditional staging systems and move toward a more personalized system of establishing prognosis.

Although the AJCC staging system for differentiated thyroid cancer is widely used to stratify prognosis and guide therapeutic strategies, it is imperfect and indeed is under revision. For example, there has been debate recently about the appropriateness of dividing T1 tumors into T1a and T1b categories if essentially prognosis for all tumors < 2 cm is excellent; others have called into question using a patient age cut-point of 45 years to drive a dichotomized staging system that is unique to differentiated thyroid cancer among all cancer diagnoses [22–24]. Tumors with the same clinicopathologic stage may include multiple subsets with differences in response to treatment and overall behavior [25–28]. It is also important to note that some PTC patients initially respond to standard treatment but then suffer disease recurrence [29–32]. In this study, we demonstrated that large tumors with lymph node metastases can be associated with excellent outcomes, while aggressive PTC can be observed in patients with AJCC Stage 1 tumors. However, Tumor, Node and Metastasis (TNM) staging lacks granularity of detail with regard to demographic, clinical, pathologic, and genomic characteristics that potentially could better capture the unique behavior of individual tumors [33–35]. For instance, according to the AJCC staging system, patients who are young (age < 45 years) are deemed to have Stage 1 PTC unless they have extra-cervical (M1) disease, and therefore a uniformly favorable diagnosis. Our findings suggest that a combined risk index using both clinical and genomic factors could discriminate more accurately among those patients who have a higher risk of progression from those patients with an excellent disease outcome and all of whom have a similar AJCC stage of disease based on clinical and pathologic factors alone. Furthermore, we identified a subset of patients with AJCC Stage 3 status who in fact appear to have indolent disease and a favorable prognosis when genomic factors are added. Therefore, it might not be necessary for these patients to receive aggressive therapy with associated treatment morbidity (surgery) or toxicity (medical therapy), even though they are currently believed to have more advanced disease.

Genomic alterations have long been proposed as diagnostic markers of PTC tumorigenesis and disease progression [36–38]. Recent advances in genomic technology enable profiling unique genomic alterations (such as mutations, copy number alterations, translocations, and/or chromosomal rearrangements) of cancer drivers that can be used as therapeutic targets or biomarkers to develop a cohesive framework for individualized cancer treatment [39–41]. Technological advances have now brought the possibility of more extensive interrogation of the genome to a clinical reality. Still, many studies are limited in their analysis of a single type of genomic alteration [42]. Using multiple forms of genomic measurements, a recent study from TCGA research network demonstrated that thyroid tumors are more genetically complex than previously thought [16]. Although BRAF mutations occur frequently in PTC [43], PTC exhibits considerable heterogeneity in the constellation of alterations that drive the malignancy [16]. To date, systematic and comprehensive genomic profiling of cancers, such as the work from TCGA, has focused on defining genomic subtypes [16, 44, 45].

Assessing prognosis for patients with cancer is challenging, as multiple forms of genomic alterations are interconnected in modulating oncogenic pathways. Single forms of genomic measurement can only be expected to explain a certain percentage of variation in prognosis. In this study, we carried out a series of genome-wide, progression-free survival analyses and identified a subset of genes whose expression, non-silent mutations, and copy number alterations collectively regulated disease progression of PTC. In particular, we found PTC progression was significantly associated with up-regulation of the FOXM1 signaling pathway. FOXM1 is a transcription factor that promotes tumorigenesis [46, 47], uncontrolled cell growth, and epithelial-mesenchymal transition [48, 49]; it has been linked to the progression of ovarian cancer [50], colorectal cancer [51], triple negative breast cancer [52], non-small cell lung cancer [53], and stomach cancer [54]. Consistent with previous reports [14, 15], our findings suggest that the FOXM1 pathway also may provide therapeutic targets to prevent PTC progression.

The clinical utility of outcome predictors is determined by whether or not these factors can alter patient management in ways that ultimately improve patient outcomes. It has been well recognized that clinical and pathological factors play an important role in PTC progression, and their performance as predictors of prognosis could overlap or be independent of genomic measurements. The AJCC staging system has long remained the gold-standard used for guiding the management of PTC; other staging systems that have been developed for PTC, such as the European Organization for Research and Treatment of Cancer (EORTC) [55]; National Thyroid Cancer Treatment Cooperative Study (NTCTCS) [34]; AGES (age, grade, extent, size); AMES (age, metastases, extent and size of tumor) [56]; and MACIS (metastases, age, completeness of resection, invasion, size) similarly do not employ genetic descriptors [57, 58]. For genomic features to be incorporated in the clinical setting, they must significantly add to standard practice. Here, we demonstrate that genomic predictors complement, rather than replace, current PTC staging. Our novel integrative risk index that added genomic measurement to conventional staging factors demonstrated superior performance in identifying the subset of early stage PTC based on clinicopathologic factors that is associated with a high risk of disease progression, as well as advanced stage PTC based on clinicopathologic factors alone that is associated with near-perfect long term outcomes. Therefore, our proposed risk index model integrates clinical and pathologic factors with genomic alterations in a novel way that provides more accurate prediction of long term outcome than current clinical staging systems alone.

A recent study from TCGA research network defined PTC into two major subtypes, BRAF-like or RAS-like tumors, which have broad implications for future customized therapeutic strategies, since BRAF-like PTCs signal preferentially through MAPK, while RAS-like PTCs signal through both MAPK and PI3K [16]. These two subtypes represent over 97% of the PTC population, so additional classification might be needed to further identify the subset of patients who will need more intense treatment informed by targeting these drivers to prevent aggressive disease progression. Therefore, we applied our C+GRI model to this newly developed PTC classification system, and defined high-risk samples as BRAF-like or RAS-like, which potentially could provide an opportunity to refine therapeutic strategies for PTC based on their genomic classification and risk of disease progression.

Our study had several limitations. First, an independent large set of thyroid tumors outside of TCGA with multiple forms of genomic measurement is not currently available as an external validation set. Therefore, we carried out an internal validation approach using 2,000 bootstrap resampling, a procedure to reduce prediction error estimation that has been applied in multiple previous studies [59–65]. Another limitation of this study is that we were missing several variables that might be informative, including exposure to radiation, family history of PTC, BMI, and extrathyroidal extension. We had a relatively small number of outcome events, and this prohibited our ability to create training and testing sets. Future studies, perhaps through consortium efforts, would be required to generate a larger number of samples with sufficient follow-up time in order to establish a clinically sustainable set of factors for informing patient risk. Potential reclassification of some encapsulated variant of PTC as non-invasive follicular thyroid neoplasms with papillary-like nuclear features (NIFTP) could evolve interpretation of our findings if this revised lexicon is incorporated by TCGA [66].

To date, there has been a relative paucity of integrative risk indices that incorporate genomic factors into clinical cancer staging, with the exception of melanoma and colon cancer [42, 67]; indeed, this has not been done for differentiated thyroid cancer in a formal sense. The current study demonstrates the value of systematically incorporating genomic and clinicopathological parameters into a novel risk index model in the setting of PTC; this approach could serve as a model for application in other malignancies for which there is TCGA data.

## MATERIALS AND METHODS

### Processing of genomic data from TCGA project

Clinical information and multiple forms of genomic data for 507 papillary thyroid carcinoma (THCA) samples were obtained from TCGA (http://cancergenome.nih.gov/) in September 2015 (Supplementary Table 1). mRNA expression of 505 samples were measured by the Illumina HiSeq V2 platform, and a normalized Expectation Maximization value (RSEM) for each of the genes was extracted from TCGA (level 2). After removing genes with a call rate lower than 90%, a total of 16,025 genes were selected for examination in this study. Level 3 somatic mutation data from 430 THCA samples measured by whole genome exome sequencing were downloaded from TCGA. Recurrently detected mutations or mutated genes were defined as those mutations or mutated genes observed in three or more samples. Somatic copy number alterations (SCNA) were measured using Affymetrix Genome-Wide Human SNP Array 6.0, and level 1 CEL files were downloaded from TCGA. Germline genotypes were generated using SNP array data measured from matched blood lymphocytes or matched normal tissue. SCNAs calls were processed as we have described previously [68]. In total, SCNAs on chromosome 1–22 were successfully measured in 489 THCA samples. We determined copy number calls at each of 1,710,630 SNPs and copy number markers as homozygous deletion (CN = 0), hemizygous deletion (CN = 1), normal copy number (CN = 2), low level amplification (CN = 3), and high level amplification (CN ≥ 4). All probe coordinates were mapped to the Genome Reference Consortium GRCh37 (hg19).

### Patient population

A complete set of genomic data, such as somatic mutation, gene expression, and somatic copy number alteration data were available for 411 samples. We focused on PTC samples, and therefore dropped those samples that were not categorized as one of the histological PTC subtypes, such as classical, follicular, or tall cell variants (*n* = 6). We also dropped samples that had a history of neoadjuvant treatment (*n* = 4), and samples for which T stage was missing (*n* = 1), or there was no information regarding clinical outcome (*n* = 1). In total, 399 PTC samples were included for progression-free survival analyses. The Duke IRB considered this study exempt since patients were de-identified.

### Genomic filtering

Progression-free survival analysis was defined as new tumor events or death after initial treatment as disease progression events, and patients’ data were censored at the time of last follow-up for those individuals without a progression event. As we described previously [68, 69], a series of whole genome Cox Proportional-Hazards Regression progression-free survival analyses (COXPH) were performed to quantify the hazard ratios associated with genomic alterations and their significance when considered alongside other demographic, clinical, and pathologic variables, such as (i) patient age at diagnosis (< 45 or ≥ 45 years based on current AJCC-UIC/TNM classification [70]) at diagnosis, (ii) tumor stage, (iii) lymph node stage (0, 1, Nx), (iv) metastatic stage (0,1, Mx), (v) histologic subtypes (Classical, Follicular, Tall cell variants), (vi) gender, and (vii) history of other malignancy.

To filter the gene expression data, we used a multivariable Cox model for each gene using all clinical variables; for example,

PFS ∼ G_i_ + Clinical Variables,

for each gene expression, G_i_. We then adjusted all the *p*-values using the Benjamini-Hochberg (B-H) procedure [71], and filtered to select those with adjusted *p*-values < 0.05. A similar procedure was utilized for mutations and mutated genes data,

PFS ∼ M_i_ + Clinical Variables,

for each mutation or mutated gene, M_i_, filtering to select with *p <* 0.05. For the copy number alterations (CNAs) data, we removed regions that contained less than 5 SNPs or that were < 10 kb in length. Each region should contain at least one known gene. For the remaining regions, we then fit two nested models,

PFS ∼ R_i_ + Clinical Variables,

PFS ∼ Clinical Variables,

and tested each region R_i_ using a likelihood ratio test (LRT) [72]. The LRT *p*-values were then adjusted using the B-H procedure.

### Elastic net regression and leave-one-out cross validation

For each of the 399 patients, the remaining 398 were used to select genomic variables by elastic net regression [73]. For the remaining patients, we fit a multivariable model that included the clinical variables and all the selected genomic variables after filtering as described. The model included the clinical variables without penalty, and the genomic variables were penalized using elastic net regression. The tuning parameter for the elastic net model was selected based upon 10-fold cross-validation. Each model generated a set of coefficients for the clinical and genomic variables, and the coefficients were averaged across all genes and models.

The risk index is a linear combination of the selected covariates multiplied by the averaged coefficients from the elastic net regression [74, 75]. We used the risk index distribution to classify each patient as low, intermediate, or high risk by using the 25th and 75th percentiles as cut points. The genomic variables with sufficiently high probability to be included (cutoff point: 0.20) were selected in the final model (C+GRI), and we retained the full model with all variables with non-zero coefficients for comparative purposes. The final model is the one that only retains those genomic variables that appear in at least 20% of all of the models across subjects, as after this cut point, the frequency of appearance dropped from 23.4% to 5.8% (Supplementary Table 9). A similar modeling process, using only the clinical variables and Cox regression was used to generate a leave-one-out risk index from averaged coefficients was completed to generate a Clinical Risk Index (CRI) model as a comparison.

### Internal validation of the model

Bootstrap resampling of the Risk-Index procedure was utilized to assess stability of the Risk Index, both for the CRI model and the C+GRI model. For each of 2,000 bootstrap samples, the risk index was re-computed using the linear combination of the previously computed average coefficients. Patients were categorized into Low, Intermediate, or High risk groups based on the 25th and 75th percentiles, and a Cox model was fit using the risk index group as a covariate. For CRI and C+GRI, we computed the time-dependent AUC for 1, 3, and 5 years survival times, as well as the difference (d = AUC_C+GRI_– AUC_CRI_). For the final models, we report the bootstrap average hazard ratio, the bootstrap average difference, d, as well as the 95% confidence intervals, respectively, based on the 2.5th and 97.5th percentiles from the bootstrap distribution. We also report the final risk grouping based on the ensemble of the bootstrap results, with each subject risk characterized according to the highest frequency across categories. This procedure was repeated for both the long model and the final model, in order to determine the impact of retaining genomic factors that appeared only rarely in the leave-one-out process. For model comparison and selection, we used the Akaike Information Criterion (AIC).

### Pathway analysis

GeneSet analysis was performed using a diverse gene set libraries from Enrichr, a comprehensive gene set enrichment analysis web server [76, 77]. In order to visualize sample set enrichment of these gene-sets (enrichment level of a gene-set in a sample), we employed Gene Set Analysis (GSA) software (R package version 1.03, https://CRAN.R-project.org/package=GSA), which implements a supervised method (class labels are known before the analysis) that computes a “maxmean” summary statistic for each gene-set. Briefly, GSA computes the average of both positive and negative aspects of gene-scores (for example, fold changes) over each gene in a gene-set, and chooses the one that is larger in absolute value. Statistically signi_cant gene-sets were obtained from a Cox Proportional-Hazards model (COXPH) after accounting for the following variables: 1) gender, 2) patient age, 3) TNM staging, and 4) tumor histology.

In order to identify pathways that are associated with patient progression-free survival, we fit the following Cox regression model-

*PFS* ∼ *Pi* + *Clinical Variables*,

For each pathway *Pi* and tested two-sided null hypothesis that pathway has no effect on PFS i.e., H_0_: *Pi* = 0. The test statistic is computed from a likelihood-ratio test. Pathways with adjusted *p value* less than 0.05 are considered statistically significant.

Functional enrichment analysis of identified PTC progression associated genes was performed using Enrichr [22, 23]. We analyzed protein-protein interaction using STRING [78] networks based on protein association knowledge from databases of physical interaction and databases of curated biological pathway knowledge (MINT, HPRD, BIND, DIP, BioGRID, KEGG, Reactome, IntAct, EcoCyc, NCI-Nature Pathway Interaction Database, GO) and predicted associations between genes based on observed patterns of simultaneous expression of genes [78].

## SUPPLEMENTARY MATERIALS FIGURES AND TABLES



## Abbreviations

AIC: Akaike Information Criterion
BH: Benjamini-Hochberg
CI: Confidence interval
CNAs: Copy number alterations
COXPH: Cox Proportional-Hazards Regression progression-free survival analyses
CRI: Clinical Risk Index
C+GRI: Clinical plus Genomic Risk Index
HR: Hazard Ration
FNA: fine needle aspiration
GSA: Gene Set Analysis
InDel: insertions or deletions of a few base pairs
LRT: likelihood ratio test
NSSM: non-silent somatic mutations
PTC: papillary thyroid cancer
RSEM: Expectation Maximization value
SCNA: Somatic copy number alterations
TCGA: The Cancer Genome Atlas
THCA: thyroid carcinoma
TNM: Tumor, Node and Metastasis staging
TSH: Thyrotropin.
