# Supplementary material for: A novel integrative risk index of papillary thyroid cancer progression combining genomic alterations and clinical factors
Source: Oncotarget. 2017 Feb 6;8(10):16690–703. doi: 10.18632/oncotarget.15128 (PMC5369994; doi:10.18632/oncotarget.15128)
Supplement: Supplementary file 1 [file oncotarget-08-16690-s001.pdf]

A novel integrative risk index of papillary thyroid cancer progression combining genomic alterations and clinical factors

Supplementary Materials

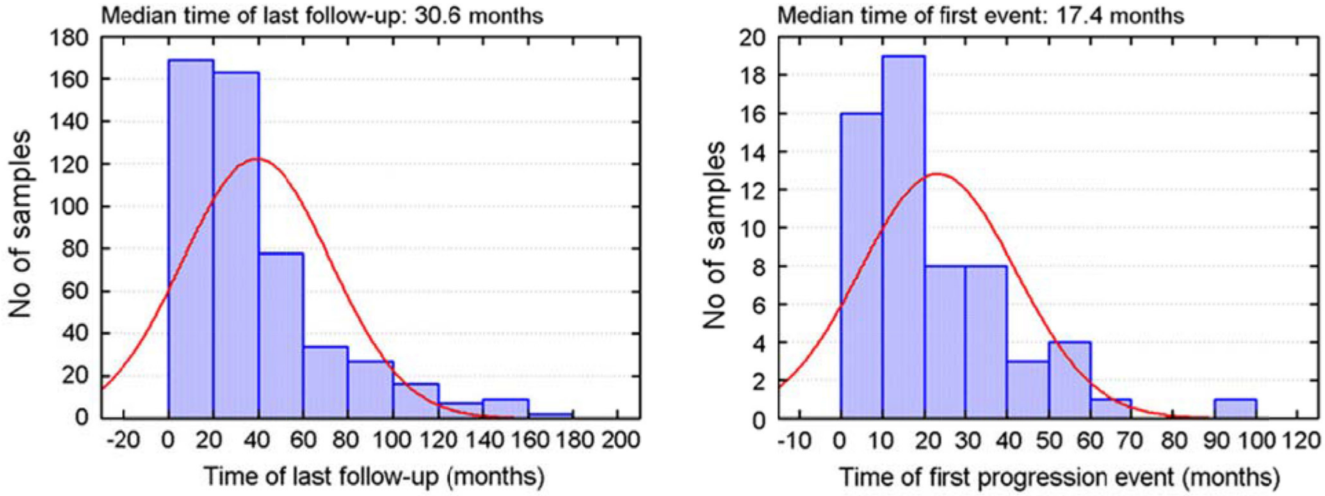

Supplementary Figure 1: Histogram of time to last follow-up and first progression event. Among 507 Thyroid Cancer samples obtained from TCGA in September 2015, the last follow-up was recorded to extend from 0 to 169.4 months. Among 60 Thyroid Cancer samples, disease progression occurred 0.2 to 97.8 months from the time of diagnosis.

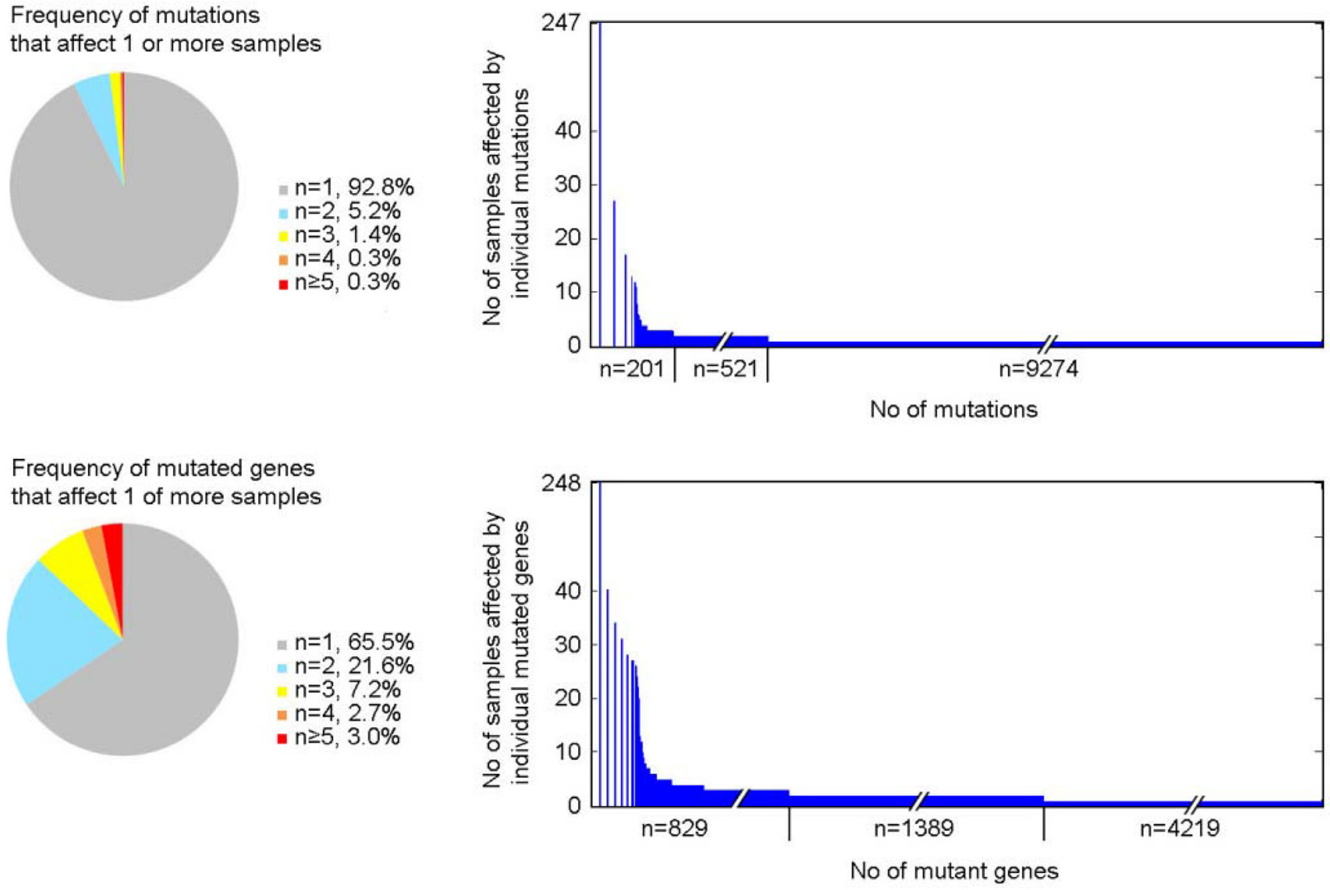

Supplementary Figure 2: Frequency of NSSM or Mutated genes that affect 1 or more samples. *Pie* figures show the percentage of samples that carry the same number of NSSMs or mutated genes among 430 Thyroid Cancer samples. *Bar* figures show number of samples that were affected by the same NSSM or mutated gene.

Supplementary Table 1: Patients' characteristics

		Number	Percentage
Age	< 45	229	45.2
	≥ 45	278	54.8
Gender	Female	371	73.2
	Male	136	26.8
Race	American Indian or Alaska Native	1	0.2
	Asian	52	10.3
	Black or African American	27	5.3
	White	333	65.7
	Not Available	94	18.5
Histological type	Classical	358	70.6
	Follicular Variant	103	20.3
	Tall	37	7.3
	Other	9	1.8
Pathologic N stage	N0	231	45.6
	N1	226	44.6
	Nx	50	9.9
Pathologic M stage	M0	283	55.8
	M1	9	1.8
	Mx	215	42.4
Pathologic T stage	T1	144	28.4
	T2	167	32.9
	T3	171	33.7
	T4	23	4.5
	Tx	2	0.4
History of other malignancy	No	473	93.3
	Yes	34	6.7

Supplementary Table 2: Genes for which expression was associated with PTC progression

Gene	*p*-value (COXPH)	BH Adjusted *p*-value*
ANLN|54443	3.61E-05	0.0297
APBA1|320	2.53E-05	0.0253
ASPM|259266	3.30E-05	0.0294
BUB1|699	4.30E-05	0.0299
CDC25C|995	4.08E-05	0.0297
CENPF|1063	5.44E-05	0.0313
CKAP2L|150468	5.01E-05	0.0309
CNTN2|6900	5.66E-05	0.0313
EPR1|8475	1.26E-05	0.0144
ESCO2|157570	7.91E-05	0.0373
EXO1|9156	6.04E-05	0.0322
EZH2|2146	2.43E-06	0.0081
F12|2161	7.02E-05	0.0352
FAM64A|54478	5.59E-06	0.0100
FOXM1|2305	2.16E-06	0.0081
HIST2H2BF|440689	3.97E-05	0.0297
HMMR|3161	1.18E-05	0.0144
KIF18B|146909	6.24E-05	0.0322
KIF20A|10112	9.82E-05	0.0425
KIF4A|24137	1.14E-05	0.0144
KIFC1|3833	9.06E-05	0.0403
MYH3|4621	4.50E-06	0.0100
NCAPG|64151	4.47E-05	0.0299
NCAPH|23397	5.65E-05	0.0313
NCRNA00176|284739	7.64E-05	0.0371
NEIL3|55247	4.97E-06	0.0100
PBK|55872	8.29E-06	0.0121
PRC1|9055	2.53E-05	0.0253
RACGAP1|29127	8.94E-05	0.0403
RRM2|6241	2.88E-05	0.0271
SKA1|220134	3.83E-05	0.0297
SKA3|221150	3.39E-06	0.0091
SPC24|147841	2.52E-06	0.0081
SPC25|57405	1.07E-04	0.0453
TOP2A|7153	1.05E-06	0.0081
TPX2|22974	7.49E-06	0.0120
TRIP13|9319	5.00E-05	0.0309
TTK|7272	2.39E-06	0.0081

*Benjamin-Hochberg adjusted *p*-value (see Methods).

Supplementary Table 3: Transcriptional pathways that were associated with PTC progression

Trianscription ChEA 2016 pathway term	Overlap	*p*-Adjusted	Genes
FOXM1_25889361_ChIP-Seq_OE33_AND_U2OS_Human	24/932	2.35E-29	TOP2A; RRM2; CKAP2L; NCAPG; TTK; HMMR; CDC25C; FOXM1; SKA3; NCAPH; ANLN; ASPM; TPX2; CENPF; KIF18B; RACGAP1; PRC1; KIFC1; KIF4A; PBK; FAM64A; KIF20A; BUB1; EZH2
FOXM1_23109430_ChIP-Seq_U2OS_Human	16/267	4.67E-24	TOP2A; RRM2; CKAP2L; TTK; HMMR; NCAPH; TPX2; ASPM; CENPF; KIF18B; RACGAP1; KIFC1; PRC1; FAM64A; KIF20A; SPC25
E2F4_17652178_ChIP-ChIP_JURKAT_Human	19/1002	3.65E-20	TOP2A; RRM2; TTK; HMMR; ESCO2; CDC25C; FOXM1; ANLN; ASPM; TPX2; CENPF; RACGAP1; EXO1; KIFC1; KIF4A; PBK; KIF20A; TRIP13; BUB1
KDM5B_21448134_ChIP-Seq_MESCs_Mouse	19/3724	5.72E-10	TOP2A; CKAP2L; NCAPG; TTK; HMMR; ESCO2; NCAPH; TPX2; ASPM; ANLN; CENPF; KIF18B; RACGAP1; PRC1; PBK; TRIP13; KIF20A; BUB1; EZH2
MYBL2_22936984_ChIP-ChIP_MESCs_Mouse	13/2250	7.42E-07	TOP2A; RRM2; CKAP2L; HMMR; CDC25C; FOXM1; TPX2; ANLN; RACGAP1; PRC1; KIF20A; EZH2; SPC25
SOX2_18692474_ChIP-Seq_MEFs_Mouse	11/1991	1.82E-05	TOP2A; TPX2; ANLN; RRM2; RACGAP1; KIFC1; PBK; HMMR; KIF20A; BUB1; EZH2
MYC_19030024_ChIP-ChIP_MESCs_Mouse	14/3868	4.01E-05	TOP2A; RRM2; TTK; HMMR; ESCO2; NCAPH; ANLN; RACGAP1; EXO1; PBK; TRIP13; KIF20A; BUB1; EZH2
MYC_18358816_ChIP-ChIP_MESCs_Mouse	13/3413	5.80E-05	TOP2A; RRM2; CKAP2L; HMMR; TPX2; ASPM; ANLN; KIFC1; EXO1; PRC1; TRIP13; KIF20A; EZH2
E2F1_18555785_ChIP-Seq_MESCs_Mouse	14/4172	7.75E-05	RRM2; CKAP2L; ESCO2; CDC25C; TPX2; ASPM; NEIL3; RACGAP1; EXO1; PRC1; KIF20A; SPC24; EZH2; SPC25
RUNX1_22897851_ChIP-Seq_JUKARTE6-1_Human	10/200	9.70E-05	TPX2; PRC1; F12; PBK; CNTN2; TTK; TRIP13; NCAPH; SPC24; EZH2

Supplementary Table 4: Recurrently detected non-silent somatic mutations and their association with PTC progression. See Supplementary_Table_4

Supplementary Table 5: Recurrently detected mutated genes and their association with PTC progression. See Supplementary_Table_5

Supplementary Table 6: Oncologic pathways of PTC progression-associated mutated genes

	GO Term	Overlap	Score	Genes
GO_Molecular_Function	ATP-dependent DNA helicase activity (GO:0004003)	2/36	7.76	CHD3; CHD2
	histone-lysine N-methyltransferase activity (GO:0018024)	2/41	7.75	KMT2B; WHSC1L1
	calmodulin binding (GO:0005516)	3/170	6.80	MYH1; SPTAN1; TRPM4
	helicase activity (GO:0004386)	3/150	6.78	DICER1; CHD3; CHD2
	ATP binding (GO:0005524)	7/1494	5.22	MYH1; DNAH1; CHD3; PRKD1; DICER1; CHD2; TRPM4
	protein N-terminus binding (GO:0047485)	2/89	5.14	SRRM2; NCOA3
	divalent inorganic cation transmembrane transporter activity (GO:0072509)	2/154	4.27	SLC39A14; TRPM4
	C2H2 zinc finger domain binding (GO:0070742)	1/15	3.86	SRRM2
	fatty acid binding (GO:0005504)	1/26	3.75	OXER1
	snRNA binding (GO:0017069)	1/18	3.61	CCNT1
	protein kinase C activity (GO:0004697)	1/16	3.60	PRKD1
	thyroid hormone receptor binding (GO:0046966)	1/27	3.47	NCOA3
GO_Biological_Process	cellular response to dsRNA (GO:0071359)	2/15	5.09	PRKRA; DICER1
	regulation of T cell cytokine production (GO:0002724)	1/15	3.82	TRPM4
	positive regulation of gene expression, epigenetic (GO:0045815)	1/16	3.79	KMT2B
	microtubule-based process (GO:0007017)	4/437	3.73	DNAH1; MAP7D2; CHD3; DICER1
	positive regulation of histone deacetylation (GO:0031065)	1/12	3.71	PRKD1
	chromatin modification (GO:0016568)	5/475	3.70	NCOA3; KMT2B; CHD3; CHD2; WHSC1L1
	reproductive structure development (GO:0048608)	3/265	3.63	NCOA3; KMT2B; DICER1
	regulation of histone modification (GO:0031056)	2/97	3.57	KMT2B; PRKD1
	actin filament capping (GO:0051693)	1/25	3.54	SPTAN1
	galactose metabolic process (GO:0006012)	1/15	3.46	GALT
	zinc ion transmembrane transport (GO:0071577)	1/15	3.34	SLC39A14
	regulation of rhodopsin mediated signaling pathway (GO:0022400)	1/30	3.16	PPEF1
GO_Cellular_component	cell-cell junction (GO:0005911)	3/335	3.98	MYH1; PRKD1; SPTAN1
	CHD-type complex (GO:0090545)	1/17	3.72	CHD3
	carboxy-terminal domain protein kinase complex (GO:0032806)	1/16	3.51	CCNT1
	axonemal dynein complex (GO:0005858)	1/12	3.50	DNAH1
	acrosomal membrane (GO:0002080)	1/13	3.49	TRIP11
	RNAi effector complex (GO:0031332)	1/10	3.37	DICER1
	Golgi apparatus (GO:0005794)	4/865	3.24	GALT; TRIP11; SLC39A14; TRPM4
	basal plasma membrane (GO:0009925)	1/26	2.99	MUC20
	Cajal body (GO:0015030)	1/30	2.96	SRRM2
	microtubule (GO:0005874)	2/360	2.46	DNAH1; MAP7D2
	methyltransferase complex (GO:0034708)	1/81	2.07	KMT2B
	cell cortex (GO:0005938)	1/83	1.88	PRKD1

Supplementary Table 7: PTC progression-associated copy number alteration regions. See Supplementary_Table_7

Supplementary Table 8: Oncologic pathways of genes within PTC progression associated copy number alteration regions

	Term	Overlap	Score	Genes
GO_Molecular_Function	interleukin-1 receptor binding (GO:0005149)	3/17	20.33	IL36B; IL36RN; IL1F10
	microfilament motor activity (GO:0000146)	2/21	8.27	MYO1B; MYO3B
	double-stranded DNA binding (GO:0003690)	3/109	6.44	SP3; RBMS1; AFF3
	NAD binding (GO:0051287)	2/50	4.24	IDH1; UXS1
	actin binding (GO:0003779)	4/386	3.84	MYO1B; FMNL2; MYO3B; PHACTR1
	growth factor binding (GO:0019838)	2/123	3.23	IL36RN; ACVR2A
	transmembrane receptor protein serine/threonine kinase activity (GO:0004675)	1/17	3.07	ACVR2A
	cAMP binding (GO:0030552)	1/23	2.98	RAPGEF4
	core promoter proximal region DNA binding (GO:0001159)	2/178	2.85	CREB1; SP3
	cation:cation antiporter activity (GO:0015491)	1/22	2.83	SLC9A4
	NAD+ binding (GO:0070403)	1/13	2.81	UXS1
	polypeptide N-acetylgalactosaminyltransferase activity (GO:0004653)	1/20	2.81	GALNT13
GO_Biological_Process	NADPH regeneration (GO:0006740)	1/13	2.75	IDH1
	sphingomyelin metabolic process (GO:0006684)	1/13	2.74	SMPD4
	natural killer cell differentiation (GO:0001779)	1/13	2.71	SP3
	response to BMP (GO:0071772)	1/22	2.68	ACVR2A
	embryonic camera-type eye morphogenesis (GO:0048596)	1/15	2.67	SP3
	stress fiber assembly (GO:0043149)	1/14	2.66	PHACTR1
	protein localization to synapse (GO:0035418)	1/14	2.65	MPP4
	cortical actin cytoskeleton organization (GO:0030866)	1/19	2.65	FMNL2
	secretory granule organization (GO:0033363)	1/19	2.63	CREB1
	cortical cytoskeleton organization (GO:0030865)	1/21	2.63	FMNL2
	embryonic skeletal system development (GO:0048706)	2/35	2.63	SP3; ACVR2A
	response to dexamethasone (GO:0071548)	1/16	2.57	MSTN
GO_Cellular_component	myosin complex (GO:0016459)	2/64	3.58	MYO1B; MYO3B
	nuclear euchromatin (GO:0005719)	1/21	3.19	CREB1
	cAMP-dependent protein kinase complex (GO:0005952)	1/9	2.99	RAPGEF4
	ciliary transition zone (GO:0035869)	1/13	2.87	TMEM237
	gamma-tubulin complex (GO:0000930)	1/15	2.84	MZT2B
	extracellular space (GO:0005615)	5/1120	2.77	MSTN; IL36B; POTEF; IL36RN; IL1F10
	chromocenter (GO:0010369)	1/13	2.77	MBD5
	synaptic membrane (GO:0097060)	2/228	2.75	DLG2; MPP4
	origin recognition complex (GO:0000808)	1/9	2.62	ORC4
	juxtaparanode region of axon (GO:0044224)	1/9	2.50	DLG2
	receptor complex (GO:0043235)	2/272	2.47	ITGB6; LRP1B
	microbody lumen (GO:0031907)	1/35	2.45	IDH1

Supplementary Table 9: Variables for risk index model*. See Supplementary_Table_9

Supplementary Table 10: Risk model using clinical variables only

Variable	Average for the Coefficients	Estimated Hazard Ratio
Age (< 45 or ≥ 45)	0.79548	2.21550
Female	–0.01992	0.98027
Classical	–0.36052	0.69731
Follicular Variant	–0.88850	0.41127
N1 stage	0.18331	1.20118
N0 stage	–0.80421	0.44744
M1 stage	1.45590	4.28834
M0 stage	0.05085	1.05216
T stage	0.16325	1.17733
History of other malignancy	0.02258	1.02283

Supplementary Table 11: Statistics for 1,3,5-years' AUC of CRI and C+GRI models

	Mean	2.5th	97.5th
**AUC_{C+GRI}_1yr**	0.73	0.67	0.789
**AUC_{C+GRI}_3yr**	0.74	0.673	0.804
**AUC_{C+GRI}_5yr**	0.724	0.656	0.793
**AUC_{CRI}_1yr**	0.676	0.607	0.743
**AUC_{CRI}_3yr**	0.683	0.614	0.753
**AUC_{CRI}_5yr**	0.667	0.597	0.738
**d_1yr**	0.054	0.009	0.107
**d_3yr**	0.057	0.008	0.111
**d_5yr**	0.057	0.009	0.111

*d_Xyr = AUC_{C+GRI}_Xyr-AUC_{CRI}_Xyr. (X = 1,3,5).
